# RhoB controls endothelial cell morphogenesis in part via negative regulation of RhoA

**DOI:** 10.1186/2045-824X-4-1

**Published:** 2012-02-08

**Authors:** Grant A Howe, Christina L Addison

**Affiliations:** 1 Program for Cancer Therapeutics, Ottawa Hospital Research Institute, Box 926, 3rd Floor TOHRCC, 501 Smyth Road, Ottawa, ON, K1H 8L6 Canada; 2 Department of Medicine, University of Ottawa, Ottawa, ON, Canada; 3 Department of Biochemistry, Microbiology and Immunology, University of Ottawa, Ottawa, ON, Canada

**Keywords:** RhoB, endothelial, RhoA, RhoC, angiogenesis, capillary morphogenesis

## Abstract

Recent studies have suggested a role for the small GTPase RhoB in the control of processes required for angiogenesis. However, the mechanisms whereby RhoB exerts control over these processes are not well understood. Given the role of vascular endothelial growth factor (VEGF) in pathological angiogenesis, we were interested in examining whether RhoB contributed to VEGF-induced angiogenic processes. To assess this, RhoB was specifically depleted in human umbilical vein endothelial cells (HUVEC), using siRNA-targeted strategies. The effects of RhoB depletion on VEGF-induced angiogenic activities were assessed using a variety of standard in vitro angiogenesis assays to assess endothelial cell viability, migration and capillary morphogenesis. Effects of RhoB depletion on signaling from other Rho family member proteins was also assessed using specific activity assays for RhoA and RhoC. We observed that although RhoB appeared dispensable for HUVEC viability, RhoB was required for endothelial cell migration, sprouting, and capillary morphogenesis. We also observed that siRNA-mediated depletion of RhoB in HUVEC resulted in increased RhoA activation in response to VEGF stimulation. This increased RhoA activation contributed to the cellular morphogenesis defects observed in RhoB-depleted cells, as inhibition of RhoA activity using C3 transferase, or inhibition of the activity of the downstream RhoA effectors Rho-dependent kinases I and II (ROCK I and II) led to a partial restoration of capillary morphogenesis in the absence of RhoB. Thus our data indicate that RhoB plays a significant role in VEGF-induced endothelial cell morphogenesis in part by negatively regulating the activity of RhoA and the RhoA/ROCK pathway.

## Background

Angiogenesis is a normal process involved in development, reproduction, and wound healing, as new blood vessels are formed from the pre-existing vasculature. Despite being a beneficial event under certain circumstances, angiogenesis is also a major contributing factor to several diseases including, rheumatoid arthritis, cancer, and ocular diseases such as diabetic retinopathy (reviewed in [1-3]). Angiogenesis is a multi-step event that requires growth factor stimulation of endothelial cells, resulting in cellular proliferation, migration, tube formation, and finally stabilization of the new vessels. As angiogenesis only initiates following angiogenic growth factor stimulation, many strategies that target primary angiogenic factors, such as vascular endothelial cell growth factor (VEGF) or its angiogenic receptor (VEGFR) have been developed and are at various stages of clinical testing. Although these types of anti-angiogenic therapies have shown some ability to control disease in certain settings, recent studies have highlighted an increased risk of severe side effects with the widely used anti-angiogenic, Bevacizumab (reviewed in [4-6]). For reasons such as this, the discovery of novel mechanisms controlling angiogenesis is necessary so that new therapeutic targets can be identified.

RhoB is a member of the Ras superfamily of GTPases, which includes proteins such as Rac1, Cdc42, RhoA, and RhoC. Rho family proteins (with the exception of RhoE) are GTPases that function by cycling through a GTP-bound activated state and a GDP-bound inactive state. Regulation of these states is achieved through GTPase-activating proteins (GAPs), guanine nucleotide exchange factors (GEFs), and guanine nucleotide dissociation inhibitors (GDIs) [7]. RhoB shares ~80% homology with its closely related family members RhoA and RhoC, however its subcellular localization was found to be very different, with almost exclusive localization to the cytosolic face of early endosomes and pre-lysosomal compartments [8]. This suggested a role in receptor trafficking, and indeed RhoB has been shown to control trafficking of a number of growth factor receptors including platelet derived growth factor receptor (PDGFR) [9], and epidermal growth factor receptor (EGFR) [10]. RhoB can contribute to growth factor receptor signaling, as it has been shown to be required for the PDGFR-driven migration of vascular smooth muscle cells via its ability to activate and traffic endosome bound Cdc42 to the cell periphery [9]. RhoB has also been shown to regulate whether EGF-bound EGFR remains in early endosomes or is transported to late endosomes for degradation, and in this manner can control duration of receptor signaling [10]. Thus, as a regulator of growth factor receptor activity, RhoB may play a significant role in mediating growth factor-induced angiogenesis.

Given its expanding role in regulating signals from angiogenic growth factor receptors, we were interested in examining the effect of RhoB on various angiogenic processes in general, and on its ability to modulate angiogenic processes induced by the primary disease-associated angiogenic factor VEGF. We hypothesized that RhoB would be required for VEGF-induced capillary morphogenesis and that the absence of RhoB would result in impaired angiogenic activities in endothelial cells. We found that VEGF stimulation upregulated expression of RhoB in endothelial cells. We also observed that although the absence of RhoB did not affect endothelial cell viability, RhoB was critically important for VEGF-induced endothelial cell migration and sprout formation. We further show that lack of RhoB in endothelial cells results in upregulation of RhoA activity, and that suppression of this activity or the activity of Rho-associated kinase (ROCK) restored VEGF-induced endothelial cell capillary morphogenesis in the absence of RhoB. We thus conclude that RhoB is required to control RhoA activity in response to VEGF stimulation to allow organization of endothelial cells during endothelial cell sprouting and capillary morphogenesis.

## Methods

### Antibodies, growth factors, and inhibitors

The following antibodies were used in this study: RhoB (C-5, SC-8048), RhoA (26 C4, SC-418), and RhoC (37, SC-130339) were all from Santa Cruz Biotechnology, Inc. (Santa Cruz, CA), monoclonal anti-β-Actin antibody (clone AC-74) (A5316; Sigma-Aldrich Co., St. Louis, MO), goat anti-mouse IgG horse radish peroxidase (HRP) conjugate (DC02L; Calbiochem, EMD Biosciences, La Jolla, CA). Recombinant human VEGF_165 _was purchased from R&D Systems (Minneapolis, MN). Cell permeable Rho Inhibitor was purchased from Cytoskeleton, Inc. (Denver, CO). ROCK I/II inhibitors H-1152 and Y-27632 were purchased from Calbiochem (EMD Biosciences, La Jolla, CA) and dissolved in dimethyl sulfoxide (DMSO).

### Cell culture

Human umbilical vein endothelial cells (HUVEC) were purchased from Lonza and passaged in EBM-2 endothelial cell basal media supplemented with EGM-2 SingleQuots, both from Lonza (Walkersville, MD), to make EGM-2 growth media. Gibco MCDB 131 was purchased from Invitrogen (Carlsbad, CA) and supplemented with L-glutamine. Where appropriate, MCDB 131 was supplemented with fetal bovine serum (FBS, Medicorp, Montreal, QC). Experiments were routinely performed with HUVEC at P6 to P10.

### siRNA transfection

For silencing of RhoB in HUVEC, two small interfering RNAs (siRNAs) were designed as ON-TARGET reagents from Dharmacon, Inc. (Lafayette, CO). Target sequences were as follows: RhoB siRNA 1: UGCUGAUCGUGUUCAGUAA, and RhoB siRNA 2: CCGUCUUCGAGAACUAUGU. Control siRNA was also purchased from Dharmacon, Inc. (siControl Non-Targeting siRNA #1; D-001210-01). For silencing experiments, RhoB siRNAs and control siRNA were used at 20 nM concentration and introduced to cells via Oligofectamine Transfection Reagent (Invitrogen, Carlsbad, CA). Cells were analyzed for protein knockdown and siRNA targeting RhoB was seen to cause maximal depletion of RhoB protein at 48 h post transfection.

### Western blotting

Western blotting was performed with NuPAGE^® ^4-12% Bis-Tris gels (Invitrogen, Carlsbad, CA). Protein detection was achieved using Immobilon Western Chemiluminescent HRP Substrate (Millipore, Billerica, MD), and images were acquired with the GeneGnome imaging system (Syngene, Frederick, MD).

### Cell viability

siRNA-transfected or mock-transfected HUVEC were seeded into 6-well tissue culture plates at 1 × 10^5 ^cells/well and sustained in EGM-2 growth media. Viability was assessed by trypan blue exclusion using a Vi-Cell XR cell viability analyzer (Beckman Coulter, Brea, CA) at the times indicated.

### Migration assay

Cell migration was assessed via scratch wound assay. Briefly, HUVEC were grown to 100% confluence and a wound of approximately 1.5 mm was made creating a gap into which cells could migrate. For siRNA experiments, wounding was performed at 48 h post transfection when RhoB depletion was maximal, and images were taken at time of wounding and 24 h post wounding with a Nikon Eclipse TE2000-U microscope. Cells were incubated in MCDB 131 with 0.05% FBS and 50 ng/ml VEGF during the course of the experiment. Percent wound closure was calculated from 12 total measurements taken across the entire wound front in duplicate dishes.

### Sprouting assay

Fibrillar collagen I gels were generated following renaturation of PureCol^® ^purified bovine dermal collagen (Advanced BioMatrix, San Diego, CA) as described by the manufacturer. Following overnight incubation to allow gels to solidify, gel surfaces were washed and briefly incubated in media prior to seeding cells at 1 × 10^5 ^cells per 6 cm dish in EGM-2 growth media supplemented with 50 ng/ml VEGF. Vessel sprouts were counted in a blinded fashion, every two days from duplicate dishes. Counts were made from 10 random fields of view per dish using an Olympus CK2 microscope. Media supplemented with VEGF was replaced every two days for the duration of the assay.

### Capillary morphogenesis

The organization of HUVEC into capillary-like networks (cords) was assessed by plating cells onto Cultrex^® ^Basement Membrane Extract (BME, Growth factor reduced, Trevigen, Gaithersburg, MD). BME was polymerized at 37 C for 30 min in 24-well plates and cells were seeded at 5 × 10^4 ^in EGM-2 growth media. Twenty-four hours later, images were taken with a Nikon Eclipse TE2000-U microscope. Demarcation of each well into quadrants allowed for a total of 4 images per well with the total number of capillary-like cords in each image counted with ImageJ software, and expressed as the average number of cords per field of view. Percent decreases were also determined following normalization of mean cord count for RhoB depleted cells to their respective control siRNA transfected cells under each condition (e.g. in absence or presence of each specific inhibitor).

### Rho activation assays

Levels of activated RhoA were determined using the RhoA G-LISA™ Activation Assay kit (Cytoskeleton Inc., Denver, CO), according to the manufacturer's instructions. Briefly, siRNA-transfected HUVEC were serum starved in MCDB 131 for 5 h at 48 h post transfection. Cells were then treated with 10 ng/ml VEGF for the times indicated and protein lysates were collected and frozen at -80 C for subsequent analysis. Protein lysates were then run on G-LISA™ plates using RhoA specific antibody for detection of captured active RhoA according to the manufacturer's directions, and absorbance was determined with a Multiskan Ascent photometer (Thermo Scientific, Rockford, IL). In all cases, constitutively active RhoA protein provided in the G-LISA™ kit, was used as a positive control to validate that the assay was functioning appropriately.

For detection of levels of active RhoC, the G-LISA™ kit was again used according to the manufacturer's instructions with the exception that a RhoC specific antibody was used to detect the amount of captured active RhoC. For the detection of RhoC, siRNA-transfected HUVEC were starved in MCDB 131 (0.5% serum) overnight, followed by starvation in serum-free MCDB 131 for 4 h. Cells were then treated with 50 ng/ml VEGF (hence stimulated 48 h post-siRNA transfection) and lysates collected 5 min post-VEGF stimulation. For the detection of activated RhoB, the G-LISA™ kit was used according to the manufacturer's instructions with the exception that a RhoB specific antibody was used to detect the amount of active RhoB captured on the plate. Activated RhoB was detected in HUVEC protein lysates collected at various times post stimulation of overnight serum starved cells (in MCDB 131 with 0.5% FBS), and their subsequent stimulation with 20 ng/ml VEGF.

## Results

RhoB has been shown to play a role in growth factor receptor trafficking and through this mechanism can regulate growth factor receptor signaling under certain circumstances [9,10]. With this in mind, we became interested in determining whether RhoB regulated VEGF-induced angiogenic processes in endothelial cells, in order to identify possible novel targets which might ultimately be useful for enhancing the efficacy of current anti-VEGF/VEGFR blocking strategies. We thus used small interfering (si)RNA silencing strategies in human umbilical vein endothelial cells (HUVEC) to determine the effects of reduced RhoB expression on the ability of VEGF to induce endothelial cell proliferation or morphogenesis, and by what potential mechanisms RhoB may regulate these angiogenic processes.

### VEGF upregulates expression of RhoB

**Figure 1:**
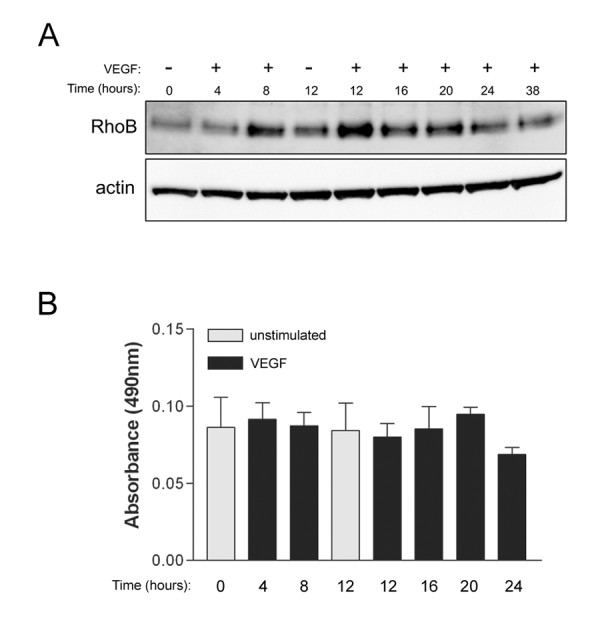
**Vascular endothelial growth factor (VEGF) stimulation results in increased expression but not activity of RhoB protein in HUVEC**. (A) RhoB protein levels are increased following VEGF stimulation. Cells were serum starved overnight in MCDB 131 with 0.25% FBS. Following this, media was replaced with MCDB 131 with 0.25% FBS and 20 ng/ml VEGF, and protein lysates were collected at the times indicated. Protein lysates were subjected to western blotting and total levels of RhoB protein were assessed. β-actin was used as a total protein loading control. (B) Levels of activated RhoB do not change following VEGF stimulation. Cells were serum starved overnight in MCDB 131 with 0.5% FBS, followed by stimulation with 20 ng/ml VEGF. At various times post-VEGF stimulation, protein lysates were collected and activated RhoB was assessed using G-LISA assays as described in Materials and Methods. Bars represent the mean ± SEM for duplicate measures in each of two independently performed experiments.

Our initial studies focused on characterizing the expression of RhoB in HUVEC as a model cell system. We found that HUVEC expressed readily detectable levels of endogenous RhoB (Figure 1A). Moreover, VEGF treatment of HUVEC resulted in increased RhoB protein levels within 8 h post-stimulation, with protein reaching maximal expression around 12 h post-VEGF stimulation (Figure 1A). To determine if increased RhoB protein levels were associated with concomitant increases in RhoB activity in these cells, we utilized the G-LISA™ Activation Assay designed for the detection of activated RhoA. This assay utilizes the Rho effector protein Rhoteckin for binding activated RhoA, and as Rhoteckin also binds RhoB, albeit much less efficiently [11], we utilized the same G-LISA™ assay but modified it for detection of the pulled-down active RhoB by using a RhoB specific monoclonal antibody for detection. Using this modified assay, we found that HUVEC do not show increased levels of active RhoB following VEGF stimulation (Figure 1B), even at time points where increased levels of RhoB protein are observed (Figure 1A).

### Depletion of RhoB levels in HUVEC inhibits cell migration but does not affect cell viability

**Figure 2:**
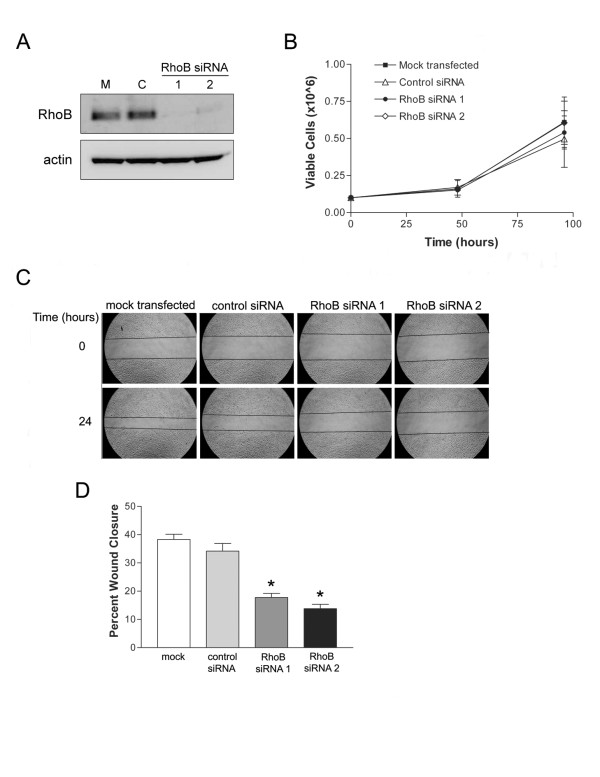
**siRNA-mediated RhoB depletion inhibits endothelial cell migration but not viability**. **(A) **Efficient depletion of RhoB protein levels was achieved in HUVEC with small interfering RNA treatment. HUVEC were either mock transfected with PBS (M) or transfected with 20 nM of the indicated siRNAs [Control (C), RhoB siRNA 1, RhoB siRNA 2]. Cellular total protein lysates were collected at 48 h post transfection and analyzed for levels of RhoB by western blot. Levels of β-actin served as a protein loading control. **(B) **Assessment of cell viability in RhoB-depleted HUVEC. Cells were either mock transfected with PBS or transfected with siRNAs, as indicated, and number of viable cells were assessed following trypan blue exclusion and counting with a Beckman Coulter Vi-Cell XR analyzer in triplicate wells at each time point. Data presented shows mean ± SD of two independently performed experiments. **(C) **RhoB-depletion inhibits cell migration. A scratch wound assay of transfected HUVEC was performed in MCDB 131, with 0.05% FBS and 50 ng/ml VEGF. Images depict wound size at time of wounding (Time = 0 h) and 24 h later. **(D) **Analysis of cell migration from (C). Bars represent the mean ± SD for percent wound closure, after 24 h for a representative experiment. *P *values were calculated using a two-tailed unpaired student's *t *test (* *P *< 0.05).

In order to assess the contribution of the small GTPase RhoB to processes important to angiogenesis, we employed a siRNA strategy to specifically deplete levels of RhoB in HUVEC. Two siRNAs directed to RhoB were validated for sequence specificity and used at 20 nM concentration to effectively reduce protein levels of RhoB in HUVEC. When compared to mock transfected and control siRNA transfected cells, there was an evident reduction in RhoB protein levels, as detected by western blot analysis, at 48 h post-siRNA transfection (Figure 2A).

As cell survival and migration are both important requirements for angiogenesis to occur, we next examined whether depleting RhoB affected either of these two processes. RhoB levels were thus depleted using two independent specific RhoB targeting siRNA constructs, and HUVEC growth and cell viability was examined over time following quantification of viable cell numbers using trypan blue exclusion. We observed no significant difference in HUVEC growth or viability between RhoB-depleted and control siRNA treated cells (Figure 2B). Cell migration was also assessed using a scratch wound assay. HUVEC were transfected with either RhoB-targeted siRNA or non-targeting control siRNA at concentrations of 20 nM and subsequently, the confluent HUVEC monolayer was 'scratched' and photographed to determine wound diameter at time 0. Media was then replaced with MCDB 131 minimal media with 0.05% FBS and supplemented with 50 ng/ml VEGF, thus making migration of HUVEC essentially VEGF-dependent. Cells were then allowed to migrate and fill the wound over the course of 24 h, at which time wound diameters were re-photographed and the percent wound closure in each condition was determined. When assessed in this manner, reduced levels of RhoB resulted in significant inhibition of cell migration as indicated by decreased percent wound closure after 24 h as compared to control siRNA transfected cells (Figure 2C and 2D). Taken together, our data suggest that RhoB plays an important role in modulating VEGF-induced cell migration signals, while appearing to be dispensable for VEGF-induced proliferative signals in endothelial cells.

### siRNA-mediated depletion of RhoB reduces HUVEC capillary sprouting and morphogenesis

**Figure 3:**
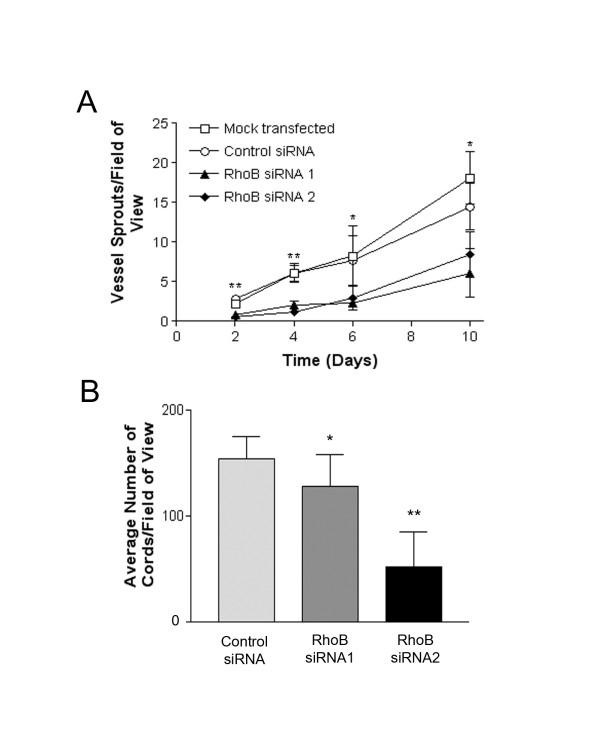
**Depletion of RhoB inhibits in vitro vessel sprouting and capillary morphogenesis**. **(A) **RhoB-depleted HUVEC have reduced ability to form vessel sprouts on collagen I gels. Cells were either mock transfected with PBS or transfected with 20 nM siRNAs (Control, RhoB siRNA 1, RhoB siRNA 2) as indicated and seeded onto collagen I gels at 24 h post transfection. At time zero (48 h post-siRNA transfection), cells were placed in EGM-2 growth media supplemented with 50 ng/ml VEGF to induce sprouting. Media supplemented with VEGF was refreshed every two days. Blinded counts were made from 10 random fields of view from each of duplicate plates, and compiled data are presented as mean ± SE of duplicate dishes from each of two independently performed experiments. Significance was determined by unpaired t-tests relative to control siRNA at each respective time point (* represents *p *< 0.05 and ** represents *p *< 0.0001) **(B) **Depletion of RhoB levels inhibit capillary morphogenesis. HUVEC were transfected with siRNAs as indicated and seeded onto polymerized basement membrane extract (BME) in EGM-2 growth media. Images were taken 24 h after cell seeding and capillary morphogenesis was determined by averaging the total number of cord-like structures in 4 fields of view as assessed with ImageJ software. The data is presented as the mean ± SE of two independently performed experiments. Significance was determined by ANOVA with Fisher's PLSD post hoc analysis (* represents *p *< 0.05 and ** represents *p *< 0.0001).

**Figure 4:**
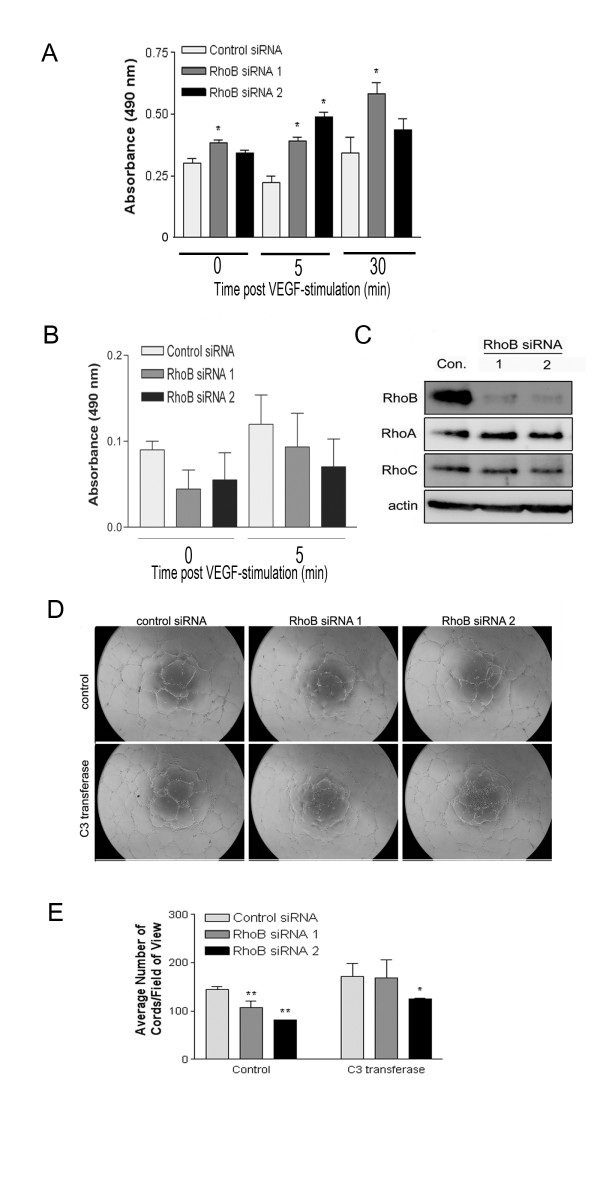
**Depletion of RhoB modulates the activity of RhoC and RhoA, and the resulting increased RhoA activity contributes to the observed reduction in capillary morphogenesis**. **(A) **G-LISA analysis indicating increased levels of activated RhoA in response to VEGF in cells with depleted RhoB expression. Cells were transfected with the indicated siRNAs, and 48 h post transfection were serum starved for 5 h in MCDB 131, and subsequently stimulated with 10 ng/ml VEGF in MCDB 131. At various times post-stimulation, protein lysates were generated and activated RhoA was detected by G-LISA assays as described in Materials and Methods. Bars represent the mean ± SE of a representative experiment performed in triplicate (* represents *p *< 0.05 as determined by unpaired t-tests when compared to Control siRNA samples at each time point). Similar trends were observed in independent experiments. **(B) **G-LISA analysis indicating reduced RhoC activity in cells treated with RhoB siRNAs. Cells were transfected with the indicated siRNAs (Control, RhoB siRNA 1, RhoB siRNA 2), followed by starvation overnight in MCDB 131 with reduced serum (0.5%), and for an additional 4 h in MCDB 131 (0% serum). Cells were subsequently stimulated with 50 ng/ml VEGF in MCDB 131 for 5 min and protein lysates were generated for analysis of RhoC activity (at 48 h post-siRNA transfection) by G-LISA as described in Materials and Methods. Bars indicate the mean ± SE for duplicate measures in each of two independently performed experiments. **(C) **Protein levels of RhoA and RhoC were assessed in RhoB siRNA-treated HUVEC. Cells were transfected with 20 nM of the indicated siRNAs and lysates collected at 48 h post transfection. Protein levels were analyzed by western blotting, with levels of β-actin serving as a control for protein loading. **(D) **Defective capillary morphogenesis in RhoB depleted HUVEC is restored upon inhibition of RhoA activity. HUVEC were transfected with siRNAs as indicated and seeded onto BME in EGM-2 growth media in the absence or presence of 0.25 μg/ml C3 transferase or DMSO as a vehicle control. Images are representative photos taken 24 h after cell seeding. **(E) **Analysis of capillary morphogenesis from (D). Capillary morphogenesis was determined by averaging the total number of cord-like structures in 4 fields of view in triplicate wells as assessed with ImageJ software. Bars represent the mean ± SE of duplicate wells from each of two independently performed experiments (* represents *p *< 0.05 and ** represents *p *< 0.0001 compared to control siRNA as determined by ANOVA with Fisher's PLSD post hoc testing).

To assess the importance of RhoB to HUVEC luminal vessel-like formation we first utilized a collagen gel-based assay. In this assay cells are placed onto a collagen I matrix and induced to sprout with VEGF, resulting in polarized vessel-like structures that contain lumen. RhoB was silenced in HUVECs using the targeted siRNAs, and 24 h later transfected HUVEC were plated on collagen I gels where they were subsequently stimulated with 50 ng/ml VEGF in EGM-2 growth medium. Sprout structures were then counted over a period of 10 days. We observed a statistically significant reduction in the number of vessel structures generated by RhoB siRNA-treated HUVEC when compared to cells treated with non-targeting control siRNA or mock transfected cells in response to VEGF stimulation (Figure 3A). We also assessed the ability of RhoB-depleted cells to form capillary-like networks on basement membrane extract (BME). In this assay, which is commonly used to test the angiogenic potential of endothelial cells, cells will usually elongate and align to form a network of cord structures that are devoid of lumens. When these cord structures were quantified, RhoB appeared to be required for HUVEC capillary morphogenesis in this assay, with HUVEC depleted of RhoB showing significant reduction in the number of cord structures formed as compared to control transfected cells (Figure 3B). It should be noted however, that the cord structures that did form in RhoB-depleted cells were similar in morphology to those observed in control-treated cells (Figure 4D), and could thus have formed as a result of incomplete RhoB depletion in 100% of cells.

### HUVEC depleted of RhoB show increased levels of activated RhoA in response to VEGF treatment

As the primary defect we observed in RhoB-depleted HUVEC was an inability to migrate and form capillary-like structures, we focused on a role for RhoB in modulating targets that regulate these pathways. Interestingly, studies have indicated that Rho protein family members can regulate one another through various mechanisms [9,12,13]. Specifically, evidence exists for unidirectional regulation of RhoB protein stability by RhoA [14]. These facts combined with the knowledge that RhoA plays an important role in cell migration led us to test whether RhoB counter-regulated RhoA, which could thus affect downstream directed cell migration and capillary morphogenesis. In order to assess the activation status of RhoA, control or RhoB-targeted siRNA transfected HUVEC were stimulated with VEGF (10 ng/ml), and protein extracts were generated over time post-VEGF stimulation to assess RhoA activity through the G-LISA™ activation assay kit as described in materials and methods. The concentration of VEGF used in this assay has previously been shown to induce RhoA activity in HUVECs [15], and under these conditions, we observed increases in RhoA activity in control siRNA treated cells as a result of VEGF stimulation alone (Figure 4A, compare 0 min to 30 min time points). However, RhoA activation observed in RhoB-depleted cells at the same time points was significantly greater than in controls (Figure 4A). It should also be noted, that even at the 0 time point, there was a modest basal increase in RhoA activity in RhoB-depleted cells compared to control cells even in the absence of VEGF stimulation (Figure 4A), supporting our hypothesis that the presence of functional RhoB may suppress RhoA activity. In addition, we also looked at the activity of RhoC, another Rho family member that has recently been indicated to play a role in endothelial cell migration and vessel organization [16]. We once again utilized the G-LISA™ activation kit; this time modifying it for use in detection of RhoC instead of RhoA through use of a RhoC specific monoclonal antibody. Interestingly, in contrast to our observations for RhoA, RhoB siRNA-treated cells had reduced levels of active RhoC in serum starved HUVECs, although this did not quite reach statistical significance (Figure 4B). We also evaluated the total protein levels of RhoA and RhoC by western blot analysis and observed no significant changes in their expression levels in HUVEC that were clearly depleted of RhoB by two different siRNAs (Figure 4C). Hence the differences observed using the G-LISA™ are indicative of differential regulation of activity of RhoA and RhoC by RhoB expression in response to VEGF stimulation.

### Inhibiting RhoA activity can partially restore capillary morphogenesis in RhoB-depleted HUVEC

In order to determine if the increased RhoA activity seen in cells depleted of RhoB contributed to the defects in capillary morphogenesis observed in RhoB-depleted cells, we inhibited the elevated RhoA activity in RhoB depleted cells and evaluated whether capillary-like network formation could be restored under these conditions. Given that we did not see significant changes in the total levels of RhoA protein, but only its activity in RhoB-depleted cells, we did not want to alter the total levels of RhoA within the cell by using a technique such as siRNA, and thus chose to pharmacologically inhibit RhoA activity. To this end, we administered the cell permeable Rho inhibitor C3 transferase. C3 transferase is an ADP ribosyltransferase that selectively ribosylates Rho proteins, rendering them inactive. Although C3 transferase does have inhibitory function against all three of the closely related Rho family members, RhoA, RhoB, and RhoC, in the absence of RhoB (which we have depleted with siRNA), C3 transferase will primarily inhibit RhoA and RhoC (which is already suppressed under these conditions) in these cells. As seen previously, RhoB-depleted HUVEC had significantly fewer cords than control treated cells (Figure 4D and 4E). However, when siRNA-transfected HUVEC were plated on BME in the presence of C3 transferase, we observed a slight increase in the number of capillary-like cords in control siRNA-transfected cells (Figure 4D and 4E), indicating the inhibition of Rho proteins with C3 transferase may have a positive effect on cord formation in general in this particular assay system. Notably, we also observed a restoration of the ability of RhoB-depleted cells to form cord structures almost to the levels of control siRNA-treated cells in the presence of C3 transferase (Figure 4D and 4E). Specifically, HUVEC treated with RhoB siRNAs 1 and 2 show reductions in capillary cord formation of approximately 25% and 45% respectively when compared to control cells in the absence of inhibitor; however, treatment with C3 transferase essentially fully restores cord formation in RhoB siRNA 1 transfected cells and restores cord formation in RhoB siRNA 2 transfected cells to within approximately 25% of control levels. Although we cannot dismiss the possibility that a proportion of the effects seen with C3 transferase could be due to inhibition of RhoC, it seems unlikely that this contributes to the restoration of cord formation, as we have shown that RhoB-depleted HUVECs already have a lower level of activated RhoC compared to control cells. Thus these results support a mechanism by which increased RhoA activity contributes to defective capillary morphogenesis in RhoB depleted HUVEC.

### Pharmacological inhibition of rho-dependent kinases ROCK I/II in RhoB-depleted HUVEC partially restores capillary morphogenesis

**Figure 5:**
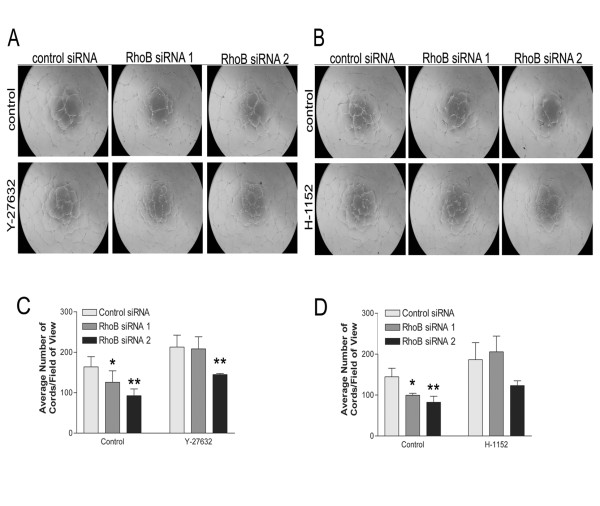
**Inhibition of ROCK partially restores capillary morphogenesis in RhoB-depleted HUVEC**. Cells were transfected with siRNAs as indicated and seeded onto BME in EGM-2 growth media in the absence or presence of **(A) **10 μM Y-27632 or **(B) **1 μM H-1152. In both cases, control media conditions contained EGM-2 growth media with equivalent volumes of DMSO as a vehicle control. Images were taken 24 h after cell seeding. **(C, D) **Analysis of capillary morphogenesis from (A, B). Capillary morphogenesis was determined by averaging the total number of cord-like structures in 4 fields of view as assessed with ImageJ software. Bars represent the mean ± SE of duplicate wells from each of two independently performed experiments (* represents p < 0.05 and ** represents p < 0.0001 as compared to the control siRNA sample using unpaired t-tests).

To further support our previous findings with the C3 transferase Rho inhibitor, we examined whether targeting pathways activated downstream of RhoA could also restore the vessel formation defects observed in RhoB-depleted cells. As the Rho-dependent kinases ROCK I and ROCK II coordinate signaling events downstream of RhoA, we targeted this pathway as a potential signaling mechanism contributing to the impaired capillary morphogenesis in RhoB-depleted cells. We thus plated control or RhoB siRNA-treated HUVEC on BME in the presence of vehicle control or two different inhibitors of ROCK I/II activity, namely Y-27632 and H-1152. Similar to what had been observed following use of C3 transferase, addition of either ROCK I/II inhibitor to control siRNA transfected cells resulted in slightly enhanced cord forming ability of HUVEC as compared to vehicle control (Figure 5A and 5C for Y-27632; Figure 5B and 5D for H-1152). Again, similar to what we had previously observed following treatment with C3 transferase, treatment of RhoB-depleted HUVEC with either ROCK inhibitor, restored cord formation in cells treated with RhoB siRNA 1 essentially to that of control siRNA levels (Figure 5C and 5D). Cord formation was also restored in RhoB siRNA 2 treated cells by the addition of ROCK inhibitors where the impairment in cord formation was reduced to only approximately 32% compared to control cells in contrast to the approximately 43% impairment in cord formation observed in the absence of ROCK inhibitors (Figure 5C and 5D). These results lend support to the notion that an inappropriately activated RhoA/ROCK pathway contributes to the observed defective capillary morphogenesis phenotype in RhoB-depleted endothelial cells.

## Discussions and conclusions

The contribution of RhoA protein signaling to processes that are important for angiogenesis, such as proliferation, migration, capillary morphogenesis and sprouting, has been previously identified (reviewed in [17]), however the contributions of the Rho family member RhoB remain less clear. Indications from knockout murine models that RhoB may modulate vessel sprouting in the retina of these mice [18], along with its proposed role in the trafficking and signaling of growth factor receptors [9,10,19,20], suggested to us that RhoB may play an important role in pathological angiogenesis directed by VEGF/VEGFR signaling. As such, RhoB could potentially prove to be an important beneficial therapeutic target for controlling pathological angiogenesis. Although some evidence suggested that RhoB could regulate endothelial cell sprouting [18], its role in growth factor induced angiogenesis was not thoroughly examined. With this in mind, the present study aimed to determine if RhoB was necessary for processes involved in VEGF-induced capillary morphogenesis, and by what mechanisms RhoB controls these events.

Our first objective was to determine whether VEGF had any direct effects on the regulation of RhoB, as certain other growth factors such as transforming growth factor β (TGF-β), and EGF have been identified as regulators of RhoB expression in a number of human cell lines [21-23]. Interestingly, we observed increased expression of RhoB protein in HUVEC following stimulation with VEGF. Results indicated a rise in protein levels between 4 and 8 h post stimulation with levels peaking at 12 h post stimulation and slowly decreasing thereafter. The mechanism leading to increased RhoB expression after VEGF treatment in HUVEC is presently unknown, and the exact implications of raising RhoB levels in VEGF stimulated HUVEC are not understood. However, it is likely that increasing RhoB expression is necessary for proper response of endothelial cells to VEGF. Indeed, transcriptional induction of the RhoB gene is achieved by TGF-β in human keratinocytes, and depletion of RhoB through siRNA has been shown to impede TGF-β-induced migration [21], indicating the potential relevance of induced expression in that system. Our finding that RhoB expression is induced by VEGF in endothelial cells highlights RhoB as a potentially important regulator of VEGF signaling, thus warranting future mechanistic studies.

In order to assess the importance of RhoB in angiogenic processes, we employed a siRNA strategy to specifically deplete HUVEC cells of RhoB, and subsequently determined whether RhoB was necessary for endothelial cell survival, migration, sprouting or capillary morphogenesis. RhoB was found to be dispensable for endothelial cell survival, as depleting RhoB levels had no effect on cell growth or viability over time. With respect to endothelial cell migration, sprouting and capillary morphogenesis, we found that RhoB was required for VEGF induction of these processes. These findings are supported by work of others in transgenic mouse or in vitro models of angiogenesis [18,24]. In contrast to the study by Sabatel et al. [24] where angiogenic activities were induced by a combination of basic fibroblast growth factor (bFGF) and VEGF together, our study focused specifically on VEGF-induced angiogenic processes. As such, our work supports a significant role for RhoB in modulating HUVEC migration and capillary morphogenesis in response to VEGF, a main mediator of angiogenesis in pathological settings.

Our results suggest that RhoB contributes to VEGF-induced endothelial cell capillary morphogenesis in part via its ability to negatively regulate RhoA. Historically, RhoA has been shown to be activated by VEGF in endothelial cells [15] and to contribute, along with other Rho family members, to the regulation of angiogenesis (reviewed in [17]). Our results now show that VEGF upregulation of RhoB plays a role in the negative regulation of RhoA activity, as when RhoB was absent, even low concentrations of VEGF induced substantial increases in RhoA activity, a phenomenon that did not occur when RhoB was present. These results thus suggest that RhoB may be important for limiting endothelial cell response to insignificant levels of VEGF that could otherwise lead to an inappropriately timed angiogenic response. Cross-regulation between Rho family members has been previously suggested. The family member Rac has been shown to regulate the activity of RhoA in fibroblasts, resulting in control of cell morphology and migration [13]. More recently, RhoA phosphorylation has been noted to release Rac from binding to RhoGDIalpha, resulting in translocation of Rac to the cell periphery followed by its activation [12]. Additionally, RhoB has been noted to traffic Cdc42 to the cell membrane in response to platelet-derived growth factor stimulation, and thus contribute to signaling necessary for cell movement [9]. Our data indicate that RhoB also negatively regulates the level of RhoA activation in response to VEGF. We further demonstrate that this inhibition of RhoA activity by RhoB is necessary for proper endothelial cell capillary morphogenesis. Moreover, activation of the RhoA/ROCK pathway has been shown to inhibit angiogenic processes [25,26], thus lending support to our observations that the absence of RhoB results in impaired angiogenic activities in part via uncontrolled RhoA/ROCK activation.

Given our results suggesting that RhoB negatively regulated RhoA activity, we were also interested to determine whether RhoB had negative effects on the activity of other Rho family members. To this end, we evaluated the effects of RhoB depletion on the level of activity of RhoC in endothelial cells. We were intrigued to observe that in contrast to our results with RhoA activation, RhoC activity was somewhat reduced in the absence of RhoB. Thus together, our results suggest that RhoB regulates the activity of RhoA and RhoC in a reciprocal manner. Although studies with RhoC-null mice did not indicate any angiogenic defects associated with primary mammary tumors [27], a more recent study showed that treatment of human dermal microvascular cells with siRNA directed to RhoC, inhibited migration and tube formation, suggesting that RhoC activity may be necessary for angiogenesis under specific conditions [16]. As RhoC can also contribute to processes such tumorigenesis and metastasis [28,29] in addition to angiogenesis [16], the RhoB regulation of RhoC activity could also be of significance in tumor growth and tumor-associated angiogenesis.

Interestingly, cross-regulation of RhoB by RhoA has been previously suggested, as it was shown that depletion of RhoA led to increased RhoGDIalpha binding to RhoB thereby resulting in RhoB protein stabilization [14]. Our study, however, demonstrates the reverse interaction, namely that RhoB negatively regulates the activation of RhoA to promote endothelial cell capillary morphogenesis. It is possible that regulation in our system is achieved via similar mechanisms as suggested by Ho et al. [14], whereby RhoA and RhoB compete for RhoGDIalpha binding, although this has not yet been demonstrated. Indeed, the importance of regulation among Rho family members mediated by binding to RhoGDIs is becoming increasingly evident, with recent studies cautioning that modulation of one Rho protein can affect others by shifting the balance of RhoGDI binding [30,31]. Additionally, it is becoming more evident that Rho proteins are spatially and temporally regulated in regards to their activity [32-34]. Evidence of a short transcript half-life for RhoB [35] indicates a high degree of regulated expression, stressing that even the induction of minor changes in RhoB expression could lead to significant effects on cell signaling. Accordingly, it is possible that when RhoB is removed, a cell can no longer control the spatially regulated activation of RhoA, resulting in delocalization of RhoA-mediated signaling events required for directed cell migration and vessel formation.

Due to their close sequence homology, RhoA and RhoB are known to bind similar protein regulators and effectors. For example, the regulator XPLN, a GEF, has been shown to specifically interact with RhoA and RhoB, but not RhoC [36]. As regulators such as GEFs and GAPs undergo translocation in response to extracellular stimuli [37,38], and in some cases to specific sites within cells occupied by their corresponding GTPase (reviewed in [7]), it is possible that competition between RhoB and RhoA for these regulators of activation is responsible for RhoB's control over RhoA activity levels in response to VEGF. Indeed, although not directly demonstrated, this has been hypothesized as a likely means of RhoB cross-regulation [39]. However, our data suggests that RhoB negatively regulates RhoA, but appears to have a positive regulatory function with respect to RhoC, suggesting that competition for activating factors or effector proteins is not the regulatory mechanism in place for this latter interaction. With this in mind, future studies will be directed towards better understanding the relationship between RhoA, RhoB, RhoC, and the various binding partners that may function to allow RhoB to regulate angiogenesis.

In conclusion, we have demonstrated that depletion of RhoB in HUVEC results in deleterious effects on processes important to angiogenesis, such as endothelial cell migration and capillary morphogenesis. These defects are, in part, due to inappropriately increased levels of activated RhoA following VEGF stimulation in the absence of RhoB. Lack of RhoC activity may also contribute to the observed defects. Based on these results we suggest a novel mechanism whereby RhoB exerts control over endothelial cell capillary morphogenesis through the negative regulation of RhoA and the positive regulation of RhoC activity in response to the pro-angiogenic growth factor VEGF.

## Abbreviations

BME: basement membrane extract; DMSO: dimethyl sulfoxide; EGF: epidermal growth factor; EGFR: epidermal growth factor receptor; FBS: fetal bovine serum; GAP: GTPase-activating protein; GDI: guanine nucleotide dissociation inhibitors; GEF: guanine nucleotide exchange factors; G-LISA: small G-protein activation assay; HRP: horse radish peroxidase; HUVEC: human umbilical vein endothelial cells; PDGFR: platelet derived growth factor receptor; ROCK: Rho-dependent kinases; siRNA: small interfering RNA; TGF-β: transforming growth factor β; VEGF: vascular endothelial growth factor; VEGFR: vascular endothelial growth factor receptor.

## Competing interests

The authors declare that they have no competing interests.

## Authors' contributions

Both authors contributed to the conception and design of experiments, acquisition, analysis and interpretation of data and were involved in drafting, critical evaluation and final approval of the manuscript.

## Author's information

^1^Program for Cancer Therapeutics, Ottawa Hospital Research Institute, Ottawa, ON, Canada.

Departments of ^2^Medicine and ^3^Biochemistry, Microbiology and Immunology, University of Ottawa, Ottawa, ON, Canada.
